# FGF1 protects FGFR1-overexpressing cancer cells against drugs targeting tubulin polymerization by activating AKT *via* two independent mechanisms

**DOI:** 10.3389/fonc.2022.1011762

**Published:** 2022-10-06

**Authors:** Jakub Szymczyk, Martyna Sochacka, Patryk Chudy, Lukasz Opalinski, Jacek Otlewski, Malgorzata Zakrzewska

**Affiliations:** Department of Protein Engineering, Faculty of Biotechnology, University of Wroclaw, Wroclaw, Poland

**Keywords:** cancer, FGF1, FGFR1, drug resistance, anticancer drugs, taltobulin, AKT

## Abstract

Cancer drug resistance is a common, unpredictable phenomenon that develops in many types of tumors, resulting in the poor efficacy of current anticancer therapies. One of the most common, and yet the most complex causes of drug resistance is a mechanism related to dysregulation of tumor cell signaling. Abnormal signal transduction in a cancer cell is often stimulated by growth factors and their receptors, including fibroblast growth factors (FGFs) and FGF receptors (FGFRs). Here, we investigated the effect of FGF1 and FGFR1 activity on the action of drugs that disrupt tubulin polymerization (taltobulin, paclitaxel, vincristine) in FGFR1-positive cell lines, U2OS stably transfected with FGFR1 (U2OSR1) and DMS114 cells. We observed that U2OSR1 cells exhibited reduced sensitivity to the tubulin-targeting drugs, compared to U2OS cells expressing a negligible level of FGFRs. This effect was dependent on receptor activation, as inhibition of FGFR1 by a specific small-molecule inhibitor (PD173074) increased the cells’ sensitivity to these drugs. Expression of functional FGFR1 in U2OS cells resulted in increased AKT phosphorylation, with no change in total AKT level. U2OSR1 cells also exhibited an elevated MDR1 and blocking MDR1 activity with cyclosporin A increased the toxicity of paclitaxel and vincristine, but not taltobulin. Analysis of tubulin polymerization pattern using fluorescence microscopy revealed that FGF1 in U2OSR1 cells partially reverses the drug-altered phenotype in paclitaxel- and vincristine-treated cells, but not in taltobulin-treated cells. Furthermore, we showed that FGF1, through activation of FGFR1, reduces caspase 3/7 activity and PARP cleavage, preventing apoptosis induced by tubulin-targeting drugs. Next, using specific kinase inhibitors, we investigated which signaling pathways are responsible for the FGF1-mediated reduction of taltobulin cytotoxicity. We found that AKT kinase is a key factor in FGF1-induced cell protection against taltobulin in U2OSR1 and DMS114 cells. Interestingly, only direct inhibition of AKT or dual-inhibition of PI3K and mTOR abolished this effect for cells treated with taltobulin. This suggests that both canonical (PI3K-dependent) and alternative (PI3K-independent) AKT-activating pathways may regulate FGF1/FGFR1-driven cancer cell survival. Our findings may contribute to the development of more effective therapies and may facilitate the prevention of drug resistance in FGFR1-positive cancer cells.

## 1 Introduction

Due to the complexity of both genetic and epigenetic factors underlying the initiation and progression of tumorigenesis, contemporary anticancer therapies are still not very effective (1). A common feature of cancer cells is their rapid and uncontrolled proliferation, often caused by overexpression of mitogenic proteins such as growth factors, or their receptors (2). Microtubules and their dynamics, involved in all phases of mitosis, are an important element in efficient cell division. Targeting tubulin, a single unit of microtubules, is one of the most common strategies used in anticancer treatment. There is a large group of drugs that act by inhibiting tubulin polymerization (e.g. vincristine) or by stabilizing the resulting microtubules and preventing their depolymerization (e.g. paclitaxel). In both cases, deregulation of tubulin polymerization leads to inhibition of cell division and tumor growth, and ultimately activates apoptosis leading to tumor cell death (3).

One of the most serious problems facing modern cancer-focused medicine is the development of drug resistance to current therapies. Mechanisms underlying chemoresistance include inhibition of apoptosis, drug inactivation, increased drug export, enhancement of DNA repair mechanisms and mutations at drug target sites (4, 5). The aforementioned growth factors, whose enhanced activity can lead to metabolic dysfunction and tumor formation, have also been implicated in the desensitization of cancer cells to drugs (6). Recently increasing attention is being paid to the fibroblast growth factors and their receptors, whose involvement in neoplasia has been demonstrated in many types of cancer (7).

The FGF family consists of 22 proteins that interact with four specific receptors (FGFR1-4) belonging to a group of receptor tyrosine kinases (RTKs). The interaction of FGFs with FGFRs leads to receptor dimerization and activation, which in turn activates signaling cascades, such as AKT/PI3K, MAPKs, PLCγ/PKC, and STATs (8). FGFR-dependent downstream signaling regulates cell differentiation, migration, apoptosis and the cell cycle, so dysregulation of the FGFR axis often leads to various systemic disorders, including cancers and the development of its drug resistance (9). The action of FGFs, particularly FGF1 and FGF2, has been correlated with chemoresistance in many types of cancer, but the exact mechanisms have not been fully described (10). Only a few studies have demonstrated an effect of FGF2 and FGFRs on paclitaxel resistance, but without clearly identifying the specific signaling pathway responsible for this phenomenon (11–13).

In the present study, by investigating the effect of drugs that interfere with tubulin polymerization in FGFR1-positive cell lines, we observed that FGF1 prevents drug-induced apoptosis. We determined that AKT kinase is a key factor in FGF1/FGFR1-dependent cell protection against tubulin-targeting drugs in U2OSR1 and DMS114 cells. This finding may be crucial in the development of more effective combination therapies for the treatment of FGFR1-positive cancers.

## 2 Materials and methods

### 2.1 Antibodies and reagents

Primary antibodies: anti-phospho-FGFR (Tyr653/Tyr654) (p-FGFR) (#06-1433) were from Merck (Darmstadt, Germany); anti-phospho-mTOR (Ser2448) (p-mTOR) (#2971), anti-FGFR1 (FGFR1) (#9740), anti-phospho-EGFR (Y1173) (p-EGFR) (#4407), anti-phospho-AKT (Ser473) (p-AKT p-S473) (#9271), anti-phospho-AKT (Thr308) (p-AKT p-T308) (#9275), anti-AKT1/2/3 (AKT) (#9272), anti-phospho-p44/42 (Thr202/Tyr204) MAP kinase (p-ERK1/2) (#9101), anti-p44/42 MAP kinase (ERK1/2) (#9102), anti-MDR1/ABCB1 (MDR1) (#12683), and anti-poly-[ADP-ribose] polymerase (PARP) (#9542) were from Cell Signaling Technology (Danvers, MA, USA); anti-mTOR (mTOR) (#T2949), anti-γ-tubulin (tubulin) (#T6557) and anti-acetylated-α-tubulin (ac-tubulin) (#T7451) were from Sigma Aldrich (St Louis, MO, USA). Horseradish peroxidase-conjugated secondary antibodies were from Jackson Immuno-Research Laboratories (Cambridge, UK) and a chemiluminescent substrate was used to visualize them in the ChemiDoc station (BioRad, Hercules, CA, USA). AlexaFluor^®^594-conjugated secondary antibodies were from Abcam (Cambridge, UK). NucBlue Live ReadyProbes Reagent was from Thermo Fisher (Waltham, MA, USA). Geneticin (G-418) was from BioShop (Puck, Poland). Penicillin-Streptomycin Solution was from Biowest (Nuaille, France). Heparin came from Sigma-Aldrich.

### 2.2 Anticancer drugs and inhibitors

Taltobulin (HTI-286) and Dactolisib (BEZ235) were from MedChem Express (Monmouth Junction, NJ, USA). Vincristine and API-2 came from Selleckchem (Houston, TX, USA). Paclitaxel, PD173074, Gefitinib, LY294002, and UO126 were purchased from Sigma-Aldrich. Torin-2 was from Cell Signaling Technology (Danvers, MA, USA), SB203580 from Calbiochem (San Diego, CA, USA), and Cyclosporin A from Carbosynth (Compton, UK).

### 2.3 Recombinant proteins

Recombinant FGF1 and FGF2 proteins were produced as previously described (14, 15). Recombinant EGF protein was obtained from M.C.Biotec.Inc (Nanjing, China).

### 2.4 Cell lines

The human osteosarcoma cell line (U2OS), and the small cell lung cancer (SCLC) cell line (DMS114) were obtained from the American Type Culture Collection (ATCC). The non-small lung cancer cell line (HCC15) was supplied by the Leibniz Institute DSMZ-German Collection of Microorganisms and Cell Cultures (DSMZ). U2OS cell lines stably transfected with pcDNA3.1 vector containing the sequence encoding the full-length FGFR1 (U2OSR1) or empty pcDNA3.1 vector (U2OS) were prepared as described previously (16). The U2OS cell line stably transfected with FGFR1-IIIc_K514R (U2OSR1-K514R) was kindly provided by Dr. Ellen M. Haugsten from the Department of Molecular Cell Biology, Institute for Cancer Research (Oslo University Hospital). U2OS, U2OSR1, and U2OSR1-K514R cells were cultured in DMEM (Biowest) supplemented with 10% fetal bovine serum (Thermo Fisher Scientific) and antibiotics (100 U/ml penicillin, 100 μg/ml streptomycin and 1 mg/ml geneticin). DMS114 cells grew in Waymouth’s MB 752/1 medium (ATCC) supplemented with 10% fetal bovine serum (Thermo Fisher Scientific) and antibiotics (100 U/mL penicillin, 100 µg/mL streptomycin). HCC15 cells were cultured in RPMI 1640 Medium (Biowest) supplemented with 10% fetal bovine serum (Thermo Fisher Scientific) and antibiotics (100 U/mL penicillin, 100 µg/mL streptomycin). All cancer cell lines were kept at 37 °C in a 5% CO_2_ incubator.

### 2.5 Cell cytotoxicity assay

Cancer cells were seeded in 96-well plates at a density of 1×10^4^ cells/well (U2OS, HCC15) or 4×10^4^ (DMS114). When comparing all three U2OS sublines for response to cytotoxic drugs, cells were kept without the addition of geneticin during the experiments. After 24 h anticancer drugs were added in various concentration (0.5 - 10 nM taltobulin (TLT), 1 - 50 nM paclitaxel (PTX) or 1 - 50 nM vincristine (VCR)) in the presence or absence of 10 ng/mL of FGF1, FGF2 or EGF and 10 U/mL heparin. When chemical inhibitors were used, they were first added to the cells for 15 min (100 nM PD174074, 20 µM UO126, 20 μM LY294002, 5 μM SB203580, 1 μM API-2, 100 nM BEZ235, 10 μM Cyclosporin A) or 60 min (100 nM Torin-2), followed by administration of the indicated drugs and/or growth factors. After 48 h of incubation, alamarBlue Cell Viability Reagent (Thermo Fisher Scientific) was added to each well according to the manufacturer’s protocol. The emission of the fluorescent reduced form of the dye was recorded at 590 nm upon excitation at 560 nm using an Infinite M1000 PRO plate reader (Tecan). The cytotoxic effect of the drugs was normalized and expressed as a percentage of cell viability of untreated cells. All experiments were performed 3 times (n=3) with at least three replicates in each experiment.

### 2.6 FGFR1 activation and downstream signaling

For the comparison of protein level and protein phosphorylation in the selected cancer cell lines, cells were seeded in 6-well plates at the density of 2×10^5^ cells/well for 24 h. Cells were lysed with sample buffer (8% SDS, 2% β-ME), and then cell lysates were sonicated, heated, and subjected to SDS-PAGE and western blotting.

For the examination of growth factors’ activity in receptor and downstream signaling activation, serum-starved (6 h) cancer cells were treated for 15 min with 100 ng/mL of FGF1, FGF2, or EGF in the presence of heparin (10 U/ml) with or without the indicated inhibitors. Inhibitors were added 15 min or 60 min (for Torin-2) before stimulation. Cells were then lysed with sample buffer (8% SDS, 2% β-ME), followed by sonication, heating, SDS-PAGE and western blotting.

### 2.7 Fluorescence microscopy

U2OSR1 cells were treated with indicated drugs with the presence or absence of 10 ng/mL FGF1 and 10 U/mL heparin for 24 h. Cells were then fixed with 4% paraformaldehyde, permeabilized with 0.1% Triton in PBS, and blocked with blocking buffer (2% BSA, 0.1 M glycine in PBS). Primary antibodies (1:500) targeted against acetylated α-tubulin were added to the cells to visualize changes in tubulin polymerization under drug conditions. Next, secondary antibodies (1:500) conjugated with AlexaFluor594 were added, followed by staining of cell nuclei with NucBlue Live ReadyProbes Reagent. Fluorescence microscopy was performed using a Zeiss Axio Observer Z1 fluorescence microscope (Zeiss, Oberkochen, Germany).

### 2.8 Cell apoptosis assays

#### 2.8.1 Caspase-3/7 activity

Cancer cells were seeded in 96-well plates at a cell density of 1×10^4^ cells/well (U2OSR1) or 4×10^4^ (DMS114). After 24 h cells were treated with indicated drugs (5 nM TLT, 20 nM PTX, or 10 nM VCR) in the presence or absence of 10 ng/mL of FGF1 and 10 U/mL heparin. 24 h later, caspases 3/7 activity was measured using the ApoLive-Glo Multiplex Assay (Promega, WI, USA) according to the manufacturer’s protocol. The ratio of caspase-3/7 activity to cell viability was normalized towards untreated cells and denoted as a relative caspase-3/7 activity. All experiments were performed three times (n=3) with at least three replicates in each experiment.

#### 2.8.2 Flow cytometry

U2OSR1 cells were seeded in 12-well plates at a cell density of 1×10^5^. After 24 h cells were treated with 5 nM TLT in the presence or absence of 10 ng/mL of FGF1 and 10 U/mL heparin. After 24 h of incubation, cells were harvested and washed with PBS. Drug-induced cell apoptosis was monitored using eBioscience™ Annexin V-FITC Apoptosis Kit (Thermo Fisher Scientific) according to the manufacturer’s protocol. Briefly, cells were first washed with binding buffer and then incubated sequentially with the indicated concentration of Annexin V-FITC and PI. Finally, all samples were analyzed using a NovoCyte 2060R Flow Cytometer (ACEA Biosciences, CA, USA), and 10,000 events were recorded for each analysis.

### 2.9 Statistical analysis

For statistical analysis, a one-tailed t-test was applied using GraphPad Prism 5 (GraphPad Software, CA, USA); p < 0.05 was considered statistically significant. The results are expressed as means ± SD.

## 3 Results

### 3.1 FGFR1 overexpression attenuates drug cytotoxicity in U2OS cells

**Figure 1:**
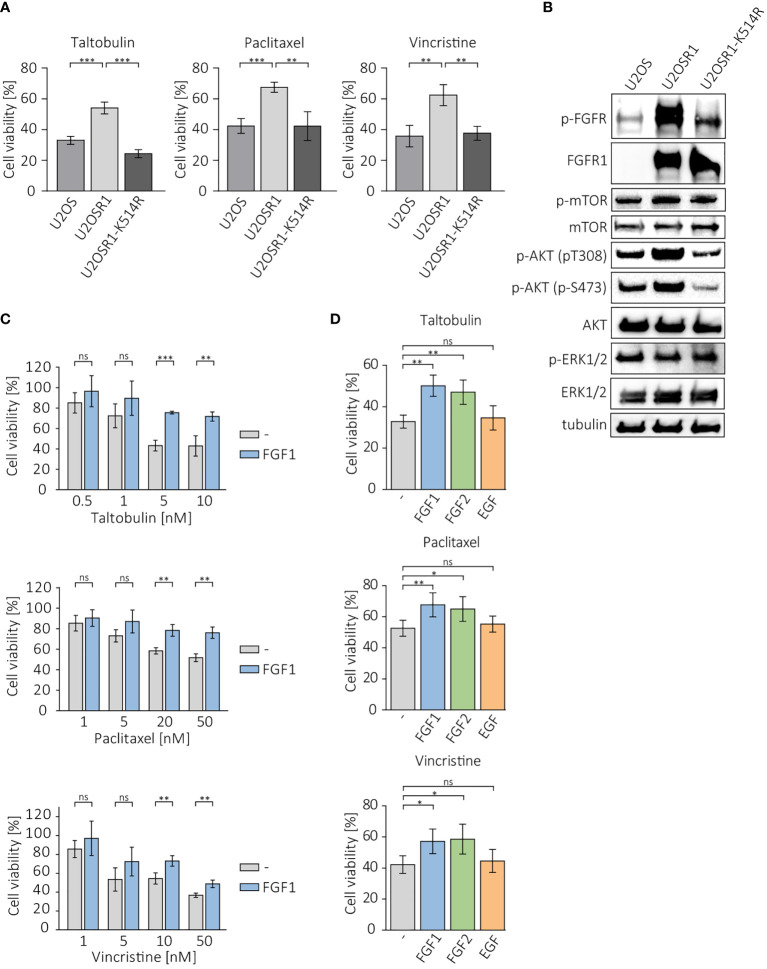
Protective effect of FGFR1 activity against cytotoxicity of taltobulin (TLT), paclitaxel (PTX) and vincristine (VCR). **(A)** Comparison of cytotoxicity of 5 nM TLT, 20 nM PTX, and 10 nM VCR in U2OS sublines. **(B)** Western blotting analysis of protein level and activation in U2OS sublines performed using the indicated primary antibodies directed to major FGFR-dependent signaling proteins. Anti-tubulin antibody served as an equal loading control. **(C)** Effect of FGF1 stimulation (10 ng/mL) in U2OSR1 cells treated with different concentration of the indicated drugs. **(D)** Effect of FGF1, FGF2, and EGF stimulation (10 ng/mL) on drug cytotoxicity in U2OSR1 cells. Cell viability in all experiments was monitored using the alamarBlue assay. Results represent the mean ± SD of at least three independent experiments and are normalized to untreated cells; statistical significance: *p<0.05, **p<0.01, ***p<0.001, no significant differences are marked as ‘ns’.

To investigate whether overproduction of FGFR1 can make cancer cells less sensitive to drugs that interfere with tubulin polymerization, we measured the viability of cells in the U2OS sublines: a control cell line, transfected with empty pcDNA3.1 vector (U2OS), cells stably transfected with FGFR1 wild-type (U2OSR1) and the kinase-dead mutant of FGFR1 (U2OSR1-K514R) after 48 h of treatment with 5 nM taltobulin (TLT), 20 nM paclitaxel (PTX), and 10 nM vincristine (VCR), using the alamarBlue Cell Viability Reagent (**Figure 1A**). For all three drugs, U2OS cells overexpressing FGFR1 (U2OSR1) show a reduced response to drug toxicity compering to U2OS cells lacking FGFR1 (U2OS). For U2OS cells overexpressing the inactive (kinase-dead) mutant of FGFR1 (U2OSR1-K514R), PTX and VCR toxicity was similar to that in control U2OS cells, and even higher for TLT.

We compared the levels and phosphorylation of major FGFR-dependent downstream signaling molecules between U2OS sublines. U2OSR1 cells showed increased phosphorylation of AKT at Ser473 and Thr308 residues without an increase in total AKT level compared to control cells or the U2OSR1-K514R line (**Figure 1B**). No differences in mTOR or ERKs activation were observed in all sublines of U2OS cells.

Next, to be able to verify the response of U2OSR1 cells treated with drugs targeting tubulin polymerization to different growth factors, we used a range of concentrations of taltobulin, paclitaxel and vincristine, estimating the drug doses at which the effect of FGF1 is most effective (**Figure 1C**). For selected concentrations (5 nM TLT, 20 nM PTX and 10 nM VCR), we analyzed the effect of stimulation of U2OSR1 cells by FGF1, FGF2 or EGF (10 ng/mL with 10 U/mL heparin). Both FGF1 and FGF2 reduced cells sensitivity to all drugs tested, while EGF stimulation had no effect on cell viability (**Figure 1D**). We observed that FGF1 protection was concentration-dependent (**Supplementary Figure 1A**). As a control, we used U2O2R1-K514R cells, in which FGF1 stimulation had no protective effect against all three drugs tested (**Supplementary Figure 1B**).

### 3.2 FGF1 and FGF2 stimulation reduces drug-induced cytotoxicity in DMS114 cancer cells

**Figure 2:**
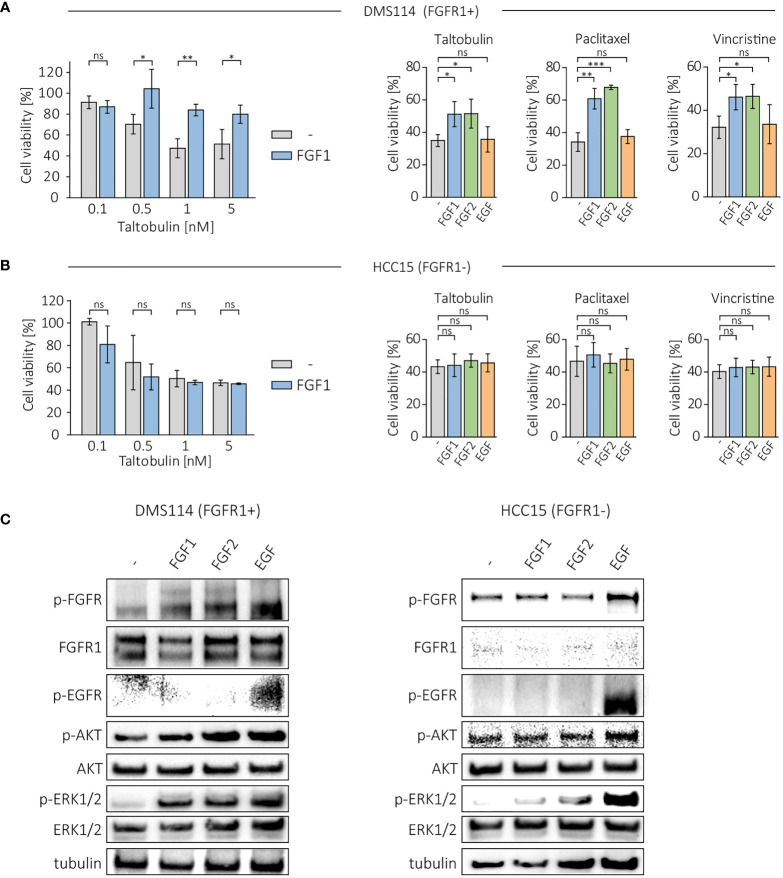
Effect of FGF1, FGF2 and EGF in DMS114 and HCC15 cells treated with the indicated drugs. Viability of **(A)** DMS114 and **(B)** HCC15 cells treated with different concentrations of TLT in the presence of 10 ng/mL FGF1 for 48 h (left panels) and comparison of the effect of FGF1, FGF2 and EGF (10 ng/mL) in cells treated for 48 h with 5 nM TLT, 20 nM PTX and 10 nM VCR (right panels). Cell viability was monitored using the alamarBlue assay. Results represent the mean ± SD of at least three independent experiments and are normalized to untreated cells; statistical significance: *p<0.05, **p<0.01, ***p<0.001, no significant differences are marked as ‘ns’. **(C)** Western blotting analysis of FGF1, FGF2 and EGF (10 ng/mL) activity in DMS114 and HCC15 cells using anti-phospho-FGFR, anti-FGFR1, anti-phospho-EGFR, anti-phospho-AKT, anti-AKT, anti-phospho-ERK1/2 and anti-ERK1/2 antibodies. Anti-tubulin antibody served as an equal loading control.

Next, we investigated the protective effect of FGF1 and FGF2 in other FGFR1-positive cancer cell lines, DMS114, and FGFR1-negative cell line, HCC15 (17, 18). Cells were treated with different concentration of the drugs (TLT, PTX and VCR) in the presence of 10 ng/mL FGF1 and 10 U/mL heparin, and cell viability was assessed after 48 h using alamarBlue assay. In DMS114 cells, the presence of FGF1 reduced the cytotoxicity of all three drugs (**Figure 2A**), while it had no in HCC15 cells (**Figure 2B**). Next, we tested the effect of FGF2 and EGF stimulation (10 ng/mL with 10 U/mL heparin) in DMS114 and HCC15 cells against the indicated drugs. FGF2 protected DMS114 cells in the same manner as FGF1 (**Figure 2A**), whereas it did not in HCC15 (**Figure 2B**). Furthermore, EGF had no effect on drugs cytotoxicity in both cancer cell lines, even in EGFR-positive HCC15 cells (**Figures 2A, B**). We also verified the short-term cell response to FGF1, FGF2 or EGF (10 ng/mL with 10 U/mL heparin) in DMS114 and HCC15 cells, by administering growth factors to serum-starved cells for 15 min and monitoring FGFR, EGFR, ERK1/2 and AKT activation by western blotting analysis (**Figure 2C**). Both FGF1 and FGF2, but not EGF, activate FGFR (upper band) in DMS114 cells. We observed a non-specific signal (lower band) detected by anti-phospho-FGFR in EGF-stimulated DMS114 cells (slightly stronger than in untreated cells) and in all HCC15 cell samples. To confirm that is not an effect of FGFR activation, we performed the experiment in both cell lines in the presence of a specific FGFR inhibitor, PD173074, and observed exactly the same pattern. However, after treatment with an EGFR inhibitor (Gefitinib), the signal was much weaker (**Supplementary Figure 2**), suggesting that the anti-phospho-FGFR antibody non-specifically recognizes some phosphorylation of EGFR. Due to the presence of both FGFR and EGFR in DMS114 cells for all growth factors, we observed ERKs phosphorylation. We observed only a slight increase in AKT phosphorylation, as even in untreated cells the level of phosphorylated AKT was relatively high. As expected, in HCC15 (FGFR-negative) cells, only EGF activated ERK1/2 and increased the level of phospho-AKT.

### 3.3 Protection against taltobulin in FGFR1-positive cells does not depend on the activity of the drug efflux proteins

**Figure 3:**
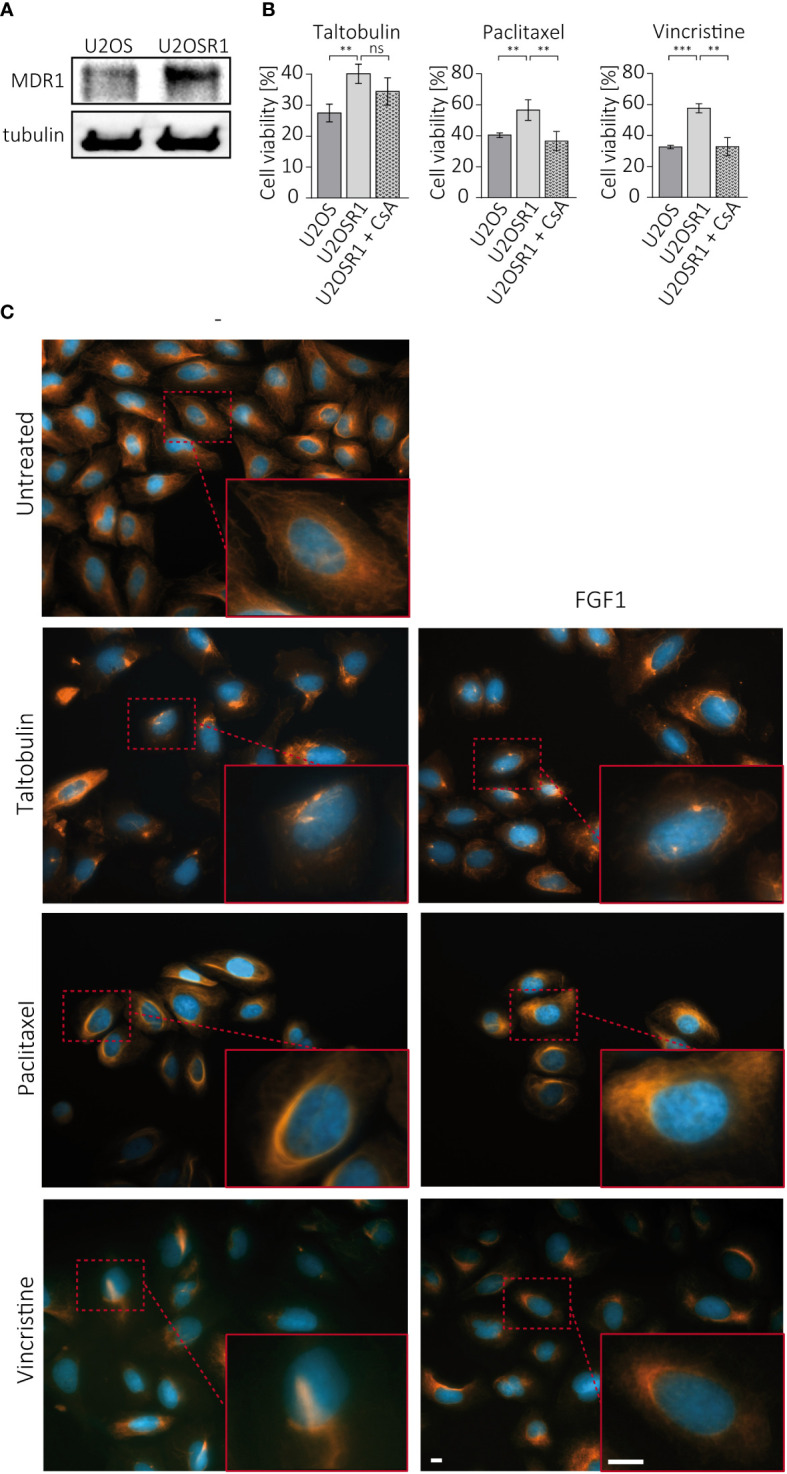
Effect of cell membrane transporters on the FGFR1-dependent protective effect against TLT, PTX and VCR. **(A)** MDR1 levels in U2OS and U2OS-R1 cells were analyzed by western blotting using anti-MDR1 antibody. **(B)** The effect of MDR1 inhibition with 10 µM cyclosporine A (CsA) on drug sensitivity in U2OSR1 cells was checked by monitoring cell viability 48 h after drug administration with the alamarBlue assay. Results represent the mean ± SD of at least three independent experiments and are normalized to untreated cells (w/o drug and CsA); statistical significance: **p<0.01, ***p<0.001, no significant differences are marked as ‘ns’. **(C)** Changes in microtubules structures after 24-h treatment with drugs (5 nM TLT, 20 nM PTX, 10 nM VCR) and the effect of FGF1 (10 ng/mL) on this process were visualized by fluorescence microscopy using antibodies against acetylated-tubulin. Scale bar represents 10 μm.

Drug resistance in cancer cells often depends on the activity of ABC transporters, which can reduce drug cytotoxicity by actively pumping toxic molecules out of the cell (2). U2OSR1 cells used in the study show high level of MDR1, one of the main efflux transporters involved in drug resistance (**Figure 3A**). To test the importance of MDR1 activity in FGFR1-dependent protection, U2OSR1 cells were treated for 1 h with cyclosporine A (CsA), an MDR1 inhibitor, and then with the indicated drugs. After 48 h, cell viability was measured in the alamarBlue assay. **Figure 3B** shows that U2OSR1 cells both untreated and treated with cyclosporine A, exhibit reduced taltobulin-induced cytotoxicity compared to U2OS control cells. However, cyclosporine A significantly lowered the protective effect of FGFR1 in U2OSR1 against paclitaxel and vincristine. We next investigated whether the FGF1 stimulation could reverse the drug-altered phenotype of tubulin polymerization in U2OSR1 cells treated with TLT, PTX or VCR. Cells were treated with the indicated drug for 24 h in the presence of 10 ng/mL FGF1 and 10 U/mL heparin. After incubation, cells were fixed and changes in tubulin polymerization were visualized with anti-acetylated-tubulin antibodies by fluorescence microscopy (**Figure 3C**). All tested drugs, according to their mechanism of action, induced changes in microtubule structure in U2OSR1 cells. However, FGF1 partially reversed the drug-altered phenotype in PTX- and VCR-treated cells, but not in TLT-treated cells. In addition, we analyzed changes in acetylated tubulin levels during incubation of U2OSR1 cells with taltobulin by western blotting. As expected, ac-tubulin levels decreased over time, but we did not observe differences due to the presence of FGF1 (**Supplementary Figure 3**). These data suggest that FGFR1-dependent signaling protects U2OSR1 cells from TLT independently of drug release from the cell, in contrast to cell protection from PTX and VCR, which is at least partially dependent on the activity of cell-membrane transporters, which may prevent drug-accumulation in cells.

### 3.4 FGF1 inhibits drug-induced apoptosis *via* activation of FGFR1

**Figure 4:**
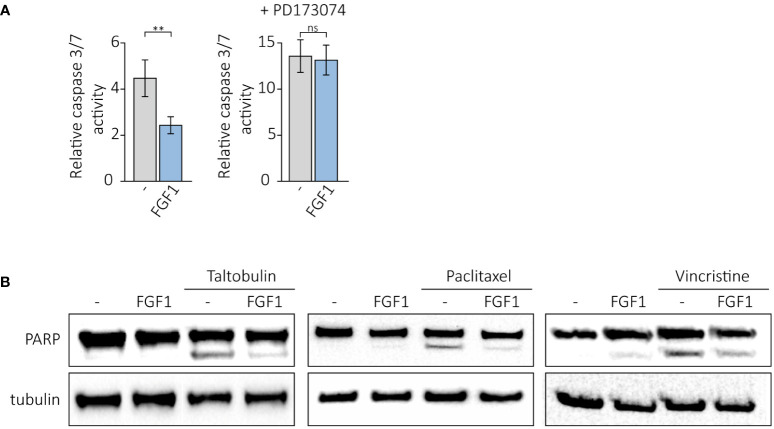
Anti-apoptotic effect of FGF1 stimulation in U2OSR1 cells treated with drugs targeting tubulin polymerization. **(A)** Relative caspase 3/7 activity induced by 5 nM TLT in U2OSR1 cells was measured using ApoLive-Glo Multiplex Assay 24 h after drug administration in the presence or absence of 10 ng/mL FGF1 and 100 nM PD173074. Results represent the mean ± SD of at least three independent experiments and are normalized to untreated cells; statistical significance: **p<0.01, no significant difference is marked as ‘ns’. **(B)** Protective effect of FGF1 against drug-induced apoptosis in U2OSR1 cells assessed by PARP cleavage. Western blotting was performed with anti-PARP antibodies 24 h after administration of 5 nM TLT, 20 nM PTX or 10 nM VCR in the presence or absence of 10 ng/mL FGF1.

We further investigated whether FGF1 stimulation could suppress drug-induced apoptosis in cancer cells expressing FGFR1. U2OSR1 cells were treated with 5 nM TLT, 10 ng/mL FGF1 and 10 U/mL heparin in the presence or absence of the potent FGFR1 inhibitor, 100 nM PD173074. After 24-h incubation, we monitored drug-induced apoptosis by measuring caspase 3/7 activity using ApoLive-Glo Multiplex Assay. FGF1 stimulation decreased relative caspase 3/7 activity in TLT-treated U2OSR1 (**Figure 4A**), and this effect was dependent on FGFR1 activation, as inhibition of the receptor kinase by PD173074 abolished it. To confirm our results, we compared the number of apoptotic and dead TLT-treated cells in the presence or absence of FGF1 by flow cytometry analysis using the eBioscience Annexin V-FITC Apoptosis Kit**(Supplementary Figure 4A**). For all three drugs (5 nM TLT, 20 nM PTX or 10 nM VCR), we also performed western blotting analysis with an anti-PARP antibody to detect PARP cleavage (**Figure 4B**). In all cases, we observed that FGF1 stimulation reduced PARP processing, demonstrating that FGF1 acts as an inhibitor of apoptosis in cancer cells treated with anticancer drugs targeting tubulin polymerization. We also confirmed the anti-apoptotic activity of FGF1 in DMS114 cells treated with TLT, PTX and VCR (**Supplementary Figure 4B**).

### 3.5 Only direct AKT inhibition abrogates the protective effect of FGF1

**Figure 5:**
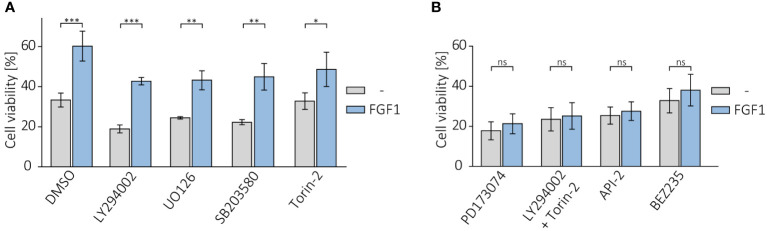
Effect of AKT inhibition on protective effect of FGF1 against TLT. Viability of U2OSR1 cells treated with 5 nM TLT and **(A)** different chemical inhibitors of major FGF-induced signaling pathways (20 µM LY294002 (PI3K), 20 µM UO126 (MEK1/2), 5 µM SB203580 (p38), 100 nM Torin-2 (mTOR)) or **(B)** FGFR inhibitor (100 nM PD173074), a direct AKT inhibitor (1 µM API-2), a dual mixture of PI3K and mTOR inhibitors (20 µM LY294002 + 100 nM Torin-2) or an inhibitor of both kinases, PI3K and mTOR (100 nM BEZ235) for 48 h in the presence or absence of 10 ng/mL FGF1, monitored by the alamarBlue assay. Results represent the mean ± SD of at least three independent experiments and are normalized to cells untreated with TLT; statistical significance: *p<0.05, **p<0.01, ***p<0.001, no significant differences are marked as ‘ns’.

Following on from our previous results, in which we showed that cells resistant to the drugs tested had an elevated level of phosphorylated AKT, we investigated the effect of inhibiting AKT and other FGFR-dependent kinases on the protective effect of FGF1. U2OS-R1 and DMS114 cells were treated with specific chemical inhibitors that block major FGFR-dependent signaling pathways (PI3K/AKT and MAPKs) known to be the main culprits of drug resistance (19–21). Cells were then treated with 5 nM TLT in the presence of 10 ng/mL FGF1 and 10 U/mL heparin. After 48 h of incubation, cell viability was monitored with alamarBlue assay. Inhibition of PI3K (upstream activator of AKT) by LY294002 or MAPK kinases by UO126 (MEK/ERKs) or by SB203580 (p38) had no effect on FGF1 action in both U2OSR1 (**Figure 5A**) and DMS114 cells (**Supplementary Figure 5A**). In contrast, inhibition of mTOR by Torin-2 partially reduced the protective effect of FGF1, especially in DMS114 cells (**Figure 5A**, **Supplementary Figure 5A**).

We next examined whether direct AKT inhibition or simultaneous blockage of both PI3K and mTOR (as an alternative activator of AKT under stress conditions) could affect the effect of FGF1 in protecting against TLT, as direct inhibition of FGFR does. Again, we confirmed that the protective effect of FGF1 stimulation was dependent on FGFR1 activation, as FGF1 did not reduce the sensitivity to TLT when FGFR was inhibited by PD173074 (**Figure 5B**). Direct inhibition of AKT with API-2 inhibitor completely abolished the protective effect of FGF1 in both U2OSR1 and DMS114 cells (**Figure 5B**, **Supplementary Figure 5B**). Moreover, the combination of PI3K and mTOR inhibitors (LY294002 and Torin-2) or a dual inhibitor of both kinases (BEZ235) also fully inhibited FGF1-induced TLT resistance (**Figure 5B**, **Supplementary Figure 5B**). This result suggests that in drug-induced cellular stress AKT can also be activated *via* a PI3K-independent pathway. Taken together, our data demonstrate that the mechanism of FGFR1’s protective effect against taltobulin cytotoxicity is directly related to AKT activation.

## 4 Discussion

The acquisition of drug resistance by cancer cells, a consequence of the enormous diversity and complexity of the molecular processes occurring in the tumor-affected tissues, results in a significant reduction in the efficacy of current anticancer therapies (1). This phenomenon affects biological drugs (such as antibodies and antibody-drug conjugates) as well as cytotoxic drugs and small-molecule inhibitors, including protein kinase inhibitors (4, 22). The two main mechanisms involved are: (i) the development of alternative pathways that transmit mitogenic signals in tumor cells bypassing the blocked molecules (23) and (ii) the overexpression of specific membrane ABC- transporters (ATP-dependent drug efflux pumps) that actively pump drugs out of the cell before they affect cell function (24, 25).

The latter mechanism is commonly observed for drugs that disrupt microtubule function and ultimately inhibit cell division, such as vinca alkaloids and taxanes (3), often leading to multidrug resistance (MDR). Other mechanisms inducing chemoresistance to tubulin-targeting drugs include mutations in the drug-binding region of β-tubulin or alterations in actin regulations (26, 27). Current knowledge of the involvement of FGFs and FGFRs in the development of cancer cell resistance to this broad group of anticancer agents is limited to a few studies describing a correlation between FGF2/FGFR1 activity and the acquisition of insensitivity to paclitaxel (12, 28, 29), most likely through stimulation of the PI3K/AKT pathway (29). There are also reports showing that inhibition of FGFRs by chemical inhibitors (such as PD173074 or BGJ398) in cancer cells increased the cytotoxicity of paclitaxel or vincristine (30–32). Undoubtedly, the actions of FGFs/FGFRs leading to cancer cell resistance include avoidance of apoptosis, EMT, stimulation of angiogenesis, and excessive cell proliferation (10). However, despite increased research into chemoresistance, the exact mechanisms activated by FGF/FGFR complexes remain unclear.

Here, we demonstrate that overexpression of FGFR1 in U2OS cells leads to reduced cytotoxicity of paclitaxel, vincristine and taltobulin compered to U2OS cells with negligibly level of FGFR1. This effect was dependent on FGFR1 activation, as it was enhanced with additional FGF1 or FGF2 stimulation and inhibited in the presence of an FGFR inhibitor. A similar effect was observed in other FGFR1-positive cells, DMS114, the small cell lung cancer cell line. In contrast, as in U2OS cells, in HCC15, an FGFR1-negative non-small lung cancer cell line, FGF1 and FGF2 did not reduce drug toxicity. Interestingly, in HCC15 cells expressing the EGF receptor, its natural ligand, EGF, did not prevent drug-induced cytotoxicity.

U2OSR1 cells showed elevated level of MDR1 protein (multidrug resistance protein 1 or P-gp), one of the most common ABC transporters involved in multidrug resistance. Blockage of MDR1 by cyclosporine A (CsA) increased sensitivity to paclitaxel and vincristine in U2OSR1 cells to the level of U2OS cells. These data suggest that overexpression of FGFR1 may affect ABC transporter levels, which in turn leads to MDR. It has already been observed that FGF2 activity correlates with increased level of MDR1 in resistant tumors (12), but to our knowledge there have been no previous unequivocal reports that FGFRs may be involved. Only two studies to date have shown that an FGFR inhibitor reverses ABC transporter-mediated MDR and restores sensitivity to paclitaxel and vincristine (31, 33). We have also demonstrated that FGF1 partially reversed the PTX- and VCR-induced alternations in microtubules structure in U2OSR1 cells, which may confirm FGFR-induced drug efflux pumps activity and reduced drug accumulation inside the treated cells.

Surprisingly, completely different results were obtained for taltobulin, a drug that inhibits tubulin polymerase very effectively (at much lower concentrations than similar drugs (34)). Firstly, cyclosporin A did not significantly affect the sensitivity of U2OSR1 cells treated with taltobulin. This may be due to the fact that taltobulin has a low affinity for ABC transporters (35), a promising feature that has led to the use of taltobulin in clinical trials for the treatment of non-small-cell lung cancer (36). Unfortunately, the phase II clinical trial was suspended before completion, and the data from this study have not been published. It has been shown that tumors can develop resistance to taltobulin trough mutations in α- or β-tubulin and through reduced accumulation of the drug in the cell regardless of the presence of MDR1 (35, 37). In agreement with these studies, we observed no differences in TLT-induced changes in microtubule structure in FGF1-stimulated cells compared to unstimulated cells, as well as in acetylated α-tubulin levels. These data suggest that the action of the FGF1/FGFR1 axis protects U2OSR1 cells independently of drug accumulation in the cells and its effect on microtubule structure. Since one mechanism of chemoresistance is apoptosis avoidance, we investigated the effect of FGF1 on drug-dependent apoptosis in U2OSR1 cells. We observed reduced caspase 3/7 activity and PARP cleavage in the presence of FGF1 in TLT-treated cells. In addition, we confirmed that FGF1 reduces PARP cleavage in U2OSR1 cells treated with PTX and VCR in the same manner as TLT. There are two main mechanisms for the anti-apoptotic action of FGF1: (i) extracellular, through activation of the receptor and initiation of downstream signaling (7), and (ii) intracellular, while FGF1, independently of receptor activation, crosses the cell membrane and interacts with apoptosis-related proteins inside the cell (38). In the case of anticancer drug resistance, the vast majority of studies describe the first mechanism as the main cause of drug insensitivity (10). However, there are reports that FGF1 can also protect cells from the effects of cisplatin and etoposide action in a receptor activation-independent manner (39, 40). In our hands, for taltobulin, this effect was completely dependent on receptor activation, as PD173074 treatment fully inhibited the anti-apoptotic activity of FGF1.

Finally, we wanted to clarify which signaling pathway(s) is responsible for the protective effect of FGF1 in cancer cells treated with taltobulin. In the last two decades of cancer drug resistance research, it has been shown that, depending on the tumor as well as the drug, different signaling pathways activated by growth factors can be crucial for reducing the efficacy of anticancer drugs (41). Thus, for example, the MAPKs pathway is responsible for tamoxifen resistance in ER-positive breast cancer (42), and the AKT pathway plays role in desensitizing EGFR-overexpressing lung cancer to gefitinib (43). Some studies have also indicated the involvement of more than one pathway in the resistance of cancer cells to drugs, suggesting the acquisition of molecular cross-talks between them (44). We therefore performed experiments using specific inhibitors of FGF/FGFR-activated major kinases in U2OSR1 and DMS114 cells treated with taltobulin. After inhibition of each of the three major signaling pathways (PI3K/AKT, ERK1/2 and p38), we observed no significant changes in the protective effect of FGF1. Inhibition of mTOR only partially reduced this effect, especially in DMS114 cells. As a next step, we decided to block AKT kinase directly, using API-2 inhibitor. In both U2OSR1 and DMS114 cells, direct AKT inhibition completely abolished the protective effect of FGF1.

Since only direct AKT inhibition affected FGF1 action in TLT-treated cells, while inhibition of the activator of this kinase (PI3K) did not, we next tested dual inhibition of PI3K and mTOR. PI3K phosphorylates AKT on the T308 residue, but further phosphorylation by mTOR on the S473 residue is required for full AKT activation (45). Only after treatment with a mixture of PI3K and mTOR inhibitors (LY294002 and Torin-2) or a dual inhibitor of both kinases (BEZ235) did we observe abrogation of the protective effect of FGF1 in TLT-treated U2OSR1 and DMS114 cells. Our observation is consistent with previous reports by Sathe and colleagues, who indicated that only simultaneous inhibition of PI3K and mTOR inhibits bladder cancer cell proliferation (46).

Interestingly, the mechanism of dual AKT activation in taltobulin protection is entirely dependent on FGFR1. No EGF-induced protective effect was observed in DMS114 or HCC15 cells, although this factor is well described as an activator of the PI3K/AKT pathway (47) and MDR gene expression (48). We suggest that AKT activation by FGFR-dependent pathway(s) in cancer cells exposed to anticancer drugs may be more complex and requires further research to fully understand it.

## Conclusions

We demonstrate for the first time that the protection of FGFR-positive cancer cells against drugs affecting tubulin polymerization is directly dependent on the action of AKT, which is activated by two alternative pathways. Only dual inhibition of PI3K and mTOR or direct blockade of AKT completely abolishes the protective effect of FGF1 against taltobulin. Our data may have important implications for understanding the mechanisms of chemoresistance and developing new combination therapy for drug-resistant tumors.

## Data availability statement

The original contributions presented in the study are included in the article/**Supplementary Material**. Further inquiries can be directed to the corresponding author.

## Author contributions

MZ designed and supervised the project. JS, LO, and MZ designed the experiments. JS, MS, PC, and LO performed the experiments. JS, MS, PC, LO, and MZ analyzed data. JS, MS, LO, and MZ prepared the figures. JS and MZ wrote the first draft of the manuscript. All authors contributed to the article and approved the submitted version.

## Funding

This work was funded by National Science Centre in Poland, grant Sonata Bis 2015/18/E/NZ3/00501. LO was supported by Sonata Bis grant 2019/34/E/NZ3/00014 from the National Science Centre, Poland.

## Acknowledgments

We thank Marta Minkiewicz for skillful assistance in cell culture. We also thank Dr. Ellen M. Haugsten from the Institute for Cancer Research (Oslo University Hospital) for providing us U2OSR1-K514R cell line.

## Conflict of interest

The authors declare that the research was conducted in the absence of any commercial or financial relationships that could be construed as a potential conflict of interest.

## Publisher's note

All claims expressed in this article are solely those of the authors and do not necessarily represent those of their affiliated organizations, or those of the publisher, the editors and the reviewers. Any product that may be evaluated in this article, or claim that may be made by its manufacturer, is not guaranteed or endorsed by the publisher.
