# Supplementary material for: FGF1 protects FGFR1-overexpressing cancer cells against drugs targeting tubulin polymerization by activating AKT via two independent mechanisms
Source: Front Oncol. 2022 Oct 6;12:1011762. doi: 10.3389/fonc.2022.1011762 (PMC9582358; doi:10.3389/fonc.2022.1011762)
Supplement: Supplementary file 1 [file DataSheet_1.pdf]

SUPPLEMENTARY FIGURES

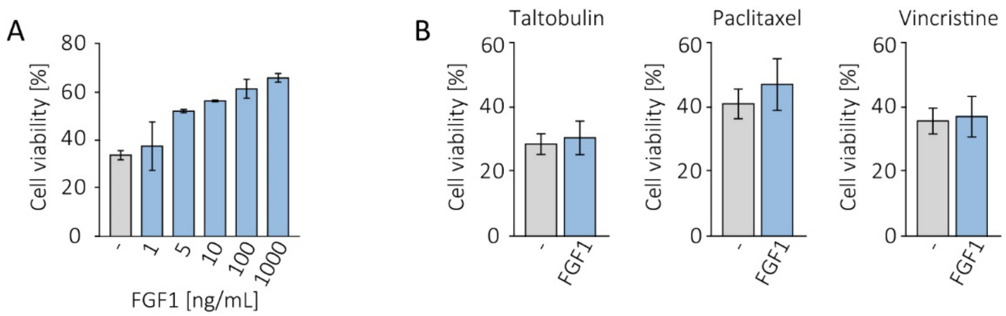

SUPPLEMENTARY FIGURE 1. (A) Protective effect of FGF1 at different concentrations against cytotoxicity of 5 nM TLT in U2OSR1 cells. **(B)** Cell viability of control U2OS cells stably transfected with kinase-dead mutant of FGFR1 (U2OSR1-K514R) treated with 5 nM TLT, 20 nM PTX or 10 nM VCR in the absence or presence of 10 ng/mL of FGF1. Cell viability was monitored using the alamarBlue assay 48 h after drug and FGF1 administration. Results represent the mean ± SD of three independent experiments and are normalized to untreated cells.

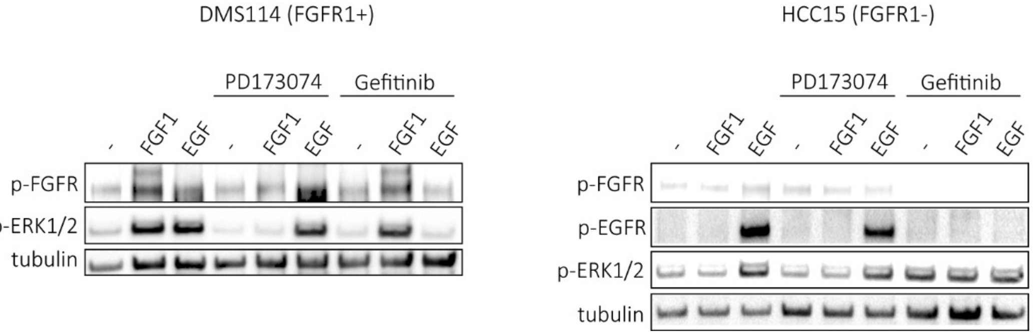

SUPPLEMENTARY FIGURE 2. Western blotting analysis of FGF1 and EGF (10 ng/mL) activity in DMS114 and HCC15 cells in the presence of specific FGFR inhibitor (100 nM PD173073) or EGFR inhibitor (10 µM Gefitinib) using anti-phospho-FGFR and anti-phospho-ERK1/2 antibodies. Anti-tubulin antibody served as an equal loading control.

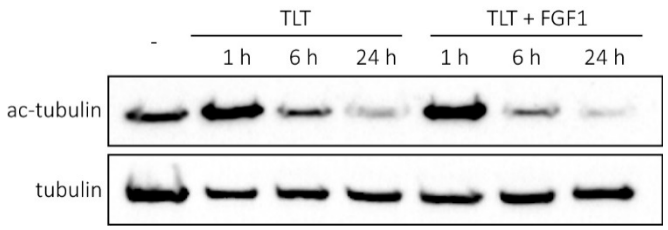

SUPPLEMENTARY FIGURE 3. Western blotting analysis of α-tubulin acetylation in U2OSR1 cells upon taltobulin-treatment (5 nM TLT) in the presence or absence of 10 ng/mL FGF1. Anti-tubulin antibody served as an equal loading control.

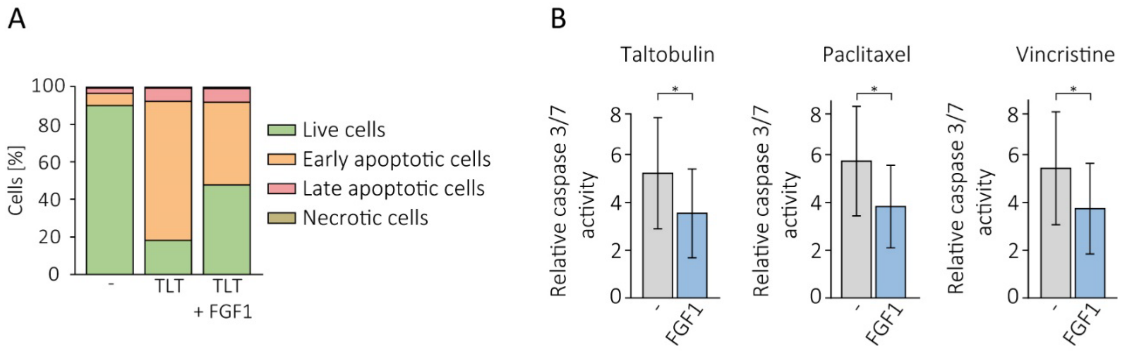

SUPPLEMENTARY FIGURE 4. Anti-apoptotic effect of FGF1 against TLT. **(A)** TLT-induced apoptosis in U2OSR1 cells measured by Annexin V and propidium iodide assay 24 h after drug administration in the presence or absence of 10 ng/mL FGF1. **(B)** Relative caspase 3/7 activity induced by 5 nM TLT, 20 nM PTX or 10 nM VCR in DMS114 cells was measured using ApoLive-Glo Multiplex Assay 24 h after drug administration in the presence or absence of 10 ng/mL FGF1 and normalized to TLT-untreated cells. Results represent the mean ± SD of three independent experiments. Results represent the mean ± SD of three independent experiments, statistical significance (paired t-test): *p<0.05

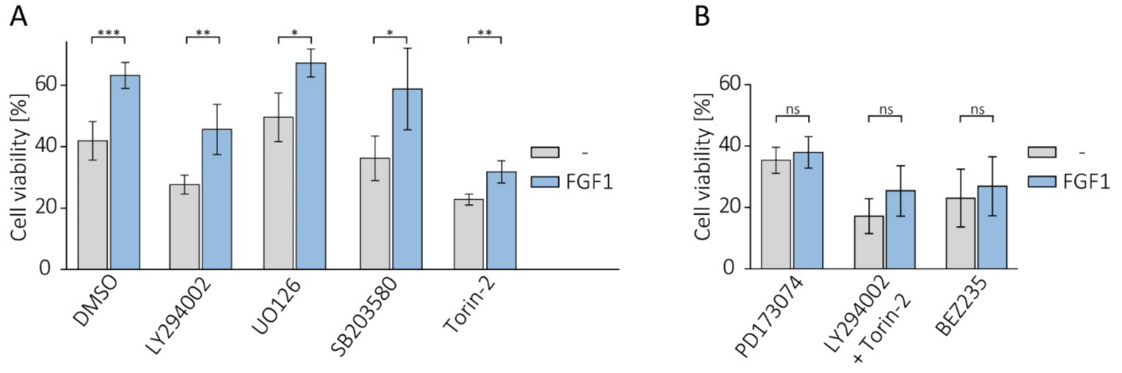

SUPPLEMENTARY FIGURE 5. Effect of AKT inhibition on the protective effect of FGF1 against TLT in DMS114 cells. Viability of cells treated with 5 nM TLT and **(A)** different chemical inhibitors of major FGF-induced signaling pathways (20 µM LY294002 (PI3K), 20 µM UO126 (MEK1/2), 5 µM SB203580 (p38), 100 nM Torin-2 (mTOR)) or **(B)** FGFR inhibitor (100 nM PD173074), a dual mixture of PI3K (20 µM LY294002) and mTOR (100 nM Torin-2) inhibitors or an inhibitor of both kinases, PI3K and mTOR (100 nM BEZ235) for 48 h in the presence or absence of 10 ng/mL FGF1, monitored by alamarBlue assay. Results are mean values ± SD of at least three independent experiments and are normalized to cells untreated with TLT; statistical significance: $*p<0.05$, $**p<0.01$, no significant differences are marked as 'ns'.
